# Mechanical Testing of Metal-Packaged FBG-Based Sensors Before and After High-Fluence Reactor Irradiation

**DOI:** 10.3390/s26144328

**Published:** 2026-07-08

**Authors:** Yerzhan Sapatayev, Kuanysh Samarkhanov, Pavel Kashaykin, Almas Azimkhanov, Sergei Vasiliev, Alexander Tomashuk, Yersin Aryngazy, Vadim Bochkov, Kamilla Ilyasheva

**Affiliations:** 1 Institute of Atomic Energy Branch of the National Nuclear Center of the Republic of Kazakhstan, 180010 Kurchatov, Kazakhstan; 2 ITER Organization, 13115 St Paul Les Durance, France; 3 Prokhorov General Physics Institute of the Russian Academy of Sciences, Dianov Fiber Optics Research Centre, 119991 Moscow, Russia; 4 School of Sciences and Humanities, Nazarbayev University, 010000 Astana, Kazakhstan

**Keywords:** FBG-based sensors, mixed gamma-neutron irradiation, post-irradiation mechanical testing, STEMET-1101 brazing filler metal

## Abstract

Fiber Bragg grating (FBG)-based sensors are increasingly used for temperature and strain monitoring in both fission and fusion facilities, whereas their long-term mechanical reliability under intense γ–neutron fields remains insufficiently understood. Although radiation-resistant FBGs and optical fibers have demonstrated tolerance to fast-neutron fluences approaching 10^20^ n/cm^2^, the post-irradiation behavior of complete sensor assemblies, including their metallic packaging and joining regions, has received much less attention. This work presents methodology and results of assessing the post-irradiation mechanical properties of packaged FBG-based temperature and strain sensors. The investigated sensors were based on Cu-coated FBGs embedded in 316L stainless-steel bodies and joined using STEMET-1101 brazing filler metal. The sensors were irradiated in the cores of the IVG.1M and WWR-K research reactors to fast-neutron fluences of 4.5 × 10^17^ and 1.8 × 10^20^ n/cm^2^, with absorbed γ-doses of 29.1 MGy and 2.3 GGy, respectively. After decay storage and hot-cell disassembly, tensile testing, microhardness measurements, and SEM–EDS analysis were performed. The results demonstrate that the investigated metal-packaged FBG sensor of this design retained mechanical integrity under high-fluence reactor irradiation.

## 1. Introduction

Optical fibers (hereafter fibers) and fiber-optic sensors based on fiber Bragg gratings (FBGs) are increasingly employed in monitoring and diagnostic systems of fission and fusion facilities. Their compact geometry, immunity to electromagnetic interference, remote interrogation capability, and stability at elevated temperatures make them suitable for harsh nuclear environments. In addition to in-core and fusion-related applications, FBG-based sensors have also been considered for spent nuclear fuel storage monitoring, including distributed water-level and temperature measurements in storage pools [1]. FBG-based sensors have also been investigated for temperature and strain monitoring in liquid-sodium environments relevant to next-generation sodium-cooled fast reactors [2]. The need for reliable monitoring technologies is also evident in spent nuclear fuel management, including the long-term dry storage of BN-350 fast reactor spent fuel, where safety assessment requires continued surveillance of sealed storage systems and their radiation conditions [3]. These examples illustrate the broad interest in fiber-optic and FBG-based sensing technologies for nuclear systems where long-term reliability of both optical and mechanical properties of the sensor is essential.

Numerous studies have demonstrated that radiation-resistant pure-silica fibers with inscribed FBGs remain functional at least up to fast-neutron fluences of 10^19^–10^20^ n/cm^2^ and γ-doses of several GGy [4,5,6,7,8,9]. Most of the previous investigations, however, were focused on radiation-induced optical effects: radiation-induced attenuation (RIA) [10], radiation-induced luminescence (RIL) [11], and radiation-induced silica compaction (RISC) [12]. Recently, Rayleigh scattering coefficient variation during mixed γ–neutron irradiation has also been reported in silica [5,13], a phenomenon referred to as neutron-induced opalescence (NIO) [13]. These studies provide an important basis for the use of FBG-based sensors in nuclear environments; however, the possible radiation-induced effects on the mechanical properties of FBG-based sensors constituted by an FBG embedded in a steel sensor body (“metal-packaged FBG-based sensor”) have virtually been ignored.

Temperature sensing in harsh environments can utilize a fiber with an FBG loosely inserted into a capillary tube without mechanical coupling with the inner capillary wall [14,15,16,17]. However, in applications requiring strain transfer, vibration monitoring, or reliable attachment to reactor components, the fiber is often mechanically coupled to the sensor body, not loosely inserted into a capillary. The possible sensor body can be metal, glass, or ceramic plates, coatings, and capillaries, which are glued, brazed, or soldered for mechanical protection and reliable operation of the FBG. Up to now, a variety of fiber packaging technologies have been developed and tested [18,19,20,21], metal packaging being the most promising approach from the standpoint of thermal stability, optical performance, and mechanical robustness. In particular, stable operation of Sn–Bi-bonded aluminum-packaged FBG-based sensors under thermal cycling and strain loading was reported in [19]. It was also shown that metal-packaged FBG-based sensors can be mechanically suitable under fatigue loading [20]. These results confirm that metal packaging can be an efficient approach to FBG-based temperature and strain sensing in harsh environments, provided that the packaged sensor assembly demonstrates robustness to reactor irradiation.

Radiation-induced or radiation-assisted changes in metal sensor components may include hardening, embrittlement, changes in local microhardness, phase-dependent degradation, porosity evolution, diffusion-zone modification, or interfacial weakening. These effects are important because, in packaged FBG-based sensors, mechanical reliability depends not only on the fiber or grating, but also on the ability of the capillary, welded regions, brazed filler metal, and stainless-steel sensor body to retain load-bearing capacity after irradiation.

In our previous in-pile experiments [4,7,22,23,24,25,26], the sensors were irradiated in the cores of IVG.1M and WWR-K reactors, at fast-neutron fluences of up to 4.5 × 10^17^ n/cm^2^ and 1.8 × 10^20^ n/cm^2^, and absorbed γ-doses of up to 29.1 MGy and 2.3 GGy, respectively. These papers described in situ and ex situ optical properties of the irradiated fibers and FBGs, including their radiation-induced optical degradation mechanisms and the spectral stability of FBGs written in radiation-resistant fibers. The same irradiation campaigns provide a valuable opportunity to ascertain whether the metal-packaged sensor design retains mechanical integrity after high-fluence reactor exposure.

The objectives of this paper are: (i) to evaluate how high-fluence reactor irradiation affects the mechanical integrity, microstructure, microhardness, and failure behavior of metal-packaged FBG-based sensors, thereby identifying the key factors that can limit their reliability in a reactor environment; and (ii) to develop and validate methodologies for post-irradiation mechanical testing of packaged FBG sensors. So, we investigate the effect of high-fluence reactor irradiation on the material and mechanical properties of FBG-based sensors consisting of Cu-coated FBGs embedded in a stainless-steel sandwich structure and brazed with STEMET-1101 brazing filler metal (BFM). Particular attention is paid to the capillary/welded attachment region and to the multiphase brazed joint, because these regions are expected to control the mechanical reliability of the packaged sensor rather than the intrinsic strength of the FBG itself.

It should be noted that radiation-induced structural changes occurring both in the metallic sensor body and in the glass fiber may affect the Bragg resonance wavelength through changes in grating length and glass density. The radiation behavior of the fibers and bare FBGs employed in these sensors has already been extensively investigated in previous publications [4,7,22,23,24,25,26,27]. Additional analysis of Bragg wavelength evolution and other optical effects in the irradiated packaged sensors will be published separately.

## 2. FBG-Based Sensor Design and Irradiation Conditions

### 2.1. Metal-Packaged FBG-Based Sensor Design

The samples were FBG-based sensors developed as prototypes for in-vessel integration in the ITER environment by the FORC-Photonics company [28]. FBG-based sensors are considered for structural health monitoring of plasma-facing and in-vessel components exposed to high thermal and mechanical loads combined with intense neutron–gamma irradiation. The sensors are intended for temperature and strain measurements of the blanket modules and divertor structures located inside the vacuum vessel. According to the ITER operational instrumentation concept, more than one thousand optical sensors are planned for installation in the vacuum vessel and divertor cassettes to provide information about thermo-mechanical loading, structural deformation, and residual lifetime of these components during reactor operation [29].

**Figure 1:**
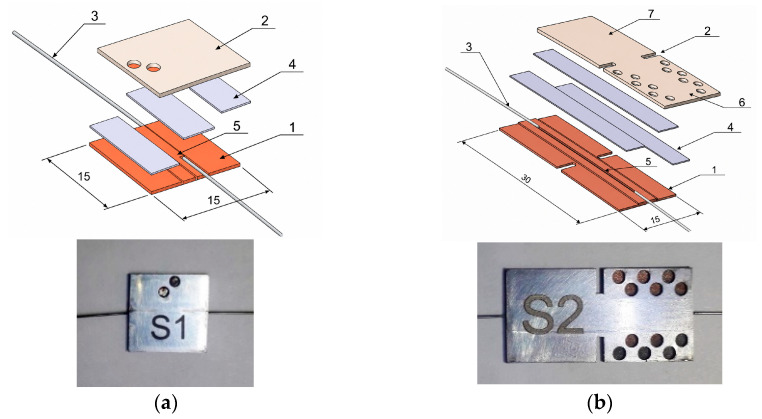
Designs (upper row) and photographs (lower row) of FBG-based sensors with a single and two FBGs. (**a**) Sensor with a single FBG: 1—lower 316L plate; 2—upper 316L plate; 3—capillary; 4—STEMET-1101 BFM, which uniformly fills the gap between the stainless-steel plates during brazing; 5—groove for fiber accommodation. (**b**) Sensor with two FBGs: 1—lower 316L plate; 2—upper 316L plate; 3—capillary; 4—STEMET-1101 BFM; 5—groove for fiber accommodation; 6—deformation part to be attached to the tested sample; 7—temperature compensation part of the sensor body.

The FBGs were written in the core of a Cu-coated pure-silica-core radiation-resistant fiber with the help of a femtosecond laser. The FBGs were brazed in a metal (316L) sensor body with STEMET-1101 BFM to provide mechanical protection of the fiber and stable strain and temperature transfer to the FBGs. Two thin steel plates formed the sensor body, with the FBG-containing fiber routed through a capillary fixed in the lower plate (Figure 1). The capillary was positioned in a machined groove and permanently attached by laser welding, providing a robust mechanical interface between the fiber and the sensor body. The overall thickness of the assembled sensor was ~1.1 mm.

The single-FBG design (15 × 15 mm) consisted of two plates clamping the fiber region with an FBG, aligned within a dedicated groove in the lower plate (Figure 1a). The dual-FBG design (15 × 30 mm) used the same sensor body concept but included two separate functional zones: a strain-sensitive region and a mechanically isolated reference zone (Figure 1b). The upper plate contained shallow recesses for positioning the tubes and two locally thinned areas intended for spot welding of the sensor to the monitored surface. The FBG region was located in the central part of the sensor and brazed between two stainless-steel plates with BFM (see Figure 1). The radiation-induced optical behavior of the fibers and FBGs was studied elsewhere [7].

During brazing, the STEMET-1101 BFM uniformly fills the gap between the stainless-steel plates around the capillary/fiber region, forming a continuous brazed layer inside the sensor body.

The most critical part of the sensor is the brazed joint, where the Cu-coated fiber enters the sensor body through the capillary. This joint is made by high-temperature STEMET-1101 BFM [30], chosen owing to its compatibility with stainless steel and good radiation resistance [31]. Since the BFM mechanical properties (porosity, intermetallic phases, adhesion, etc.) can be sensitive to radiation, we studied this region after irradiation especially carefully with mechanical and metallographic techniques.

### 2.2. Experimental Ampoule Device and Irradiation Conditions

**Figure 2:**
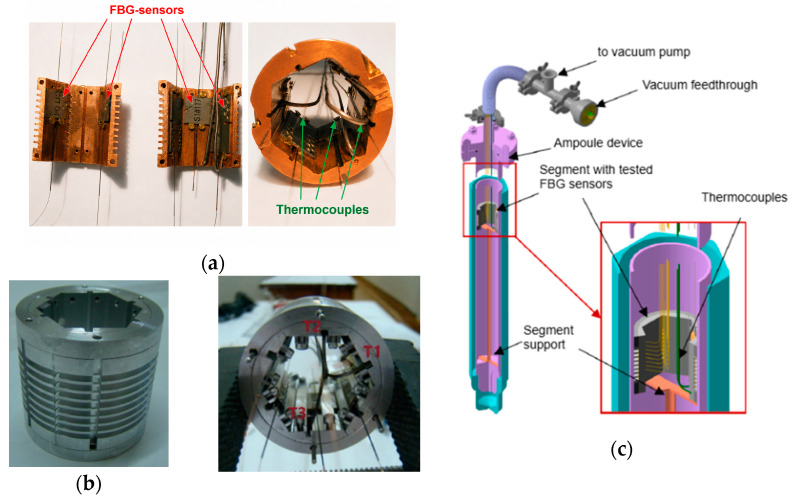
Copper segment for IVG.1M (**a**); aluminum segment for WWR-K (**b**); 3D view of the experimental ampoule device (EAD) design and the inner segment equipped with FBG-based sensors and thermocouples (**c**).

Irradiation was carried out using two identical hermetic experimental ampoule devices (EAD) previously described in [4,22]. The EAD core was constituted by a cylindrical inner segment with a hexagonal internal surface, the segment consisting of two parts to ease fixation of the sensor on its inner surface (Figure 2a,b). There were grooves on the segment external surface to wind long lengths of different fibers irradiated simultaneously with the sensors. The material of the segment was copper at the IVG.1M reactor (Figure 2a), and aluminum at the WWR-K reactor (Figure 2b), the specific material being selected according to the neutron and thermal simulation of the EAD performance [25]. After positioning the sensors on the segment’s inner surface, the two parts of the segment were assembled and tightly inserted into the EAD (Figure 2c). The EADs were vacuumized during both irradiation campaigns (Table 1).

Three K-type thermocouples were installed through hermetic electrical feedthroughs in the EAD lid to measure temperature. Two thermocouples were inserted into holes in the upper and lower regions of the inner segment to measure the vertical temperature gradient. The junction of the third thermocouple was in contact with one of the sensor bodies.

The sensors were irradiated in two reactor campaigns at IVG.1M and WWR-K research reactors [4,22]. The first irradiation [22] was performed at the IVG.1M reactor under a mixed γ–neutron field at a thermal power of 1–6 MW. The EAD used in this campaign was cooled with nitrogen to maintain the sensor temperature of ~230–330 °C. The accumulated fast-neutron fluence reached 4.5 × 10^17^ n/cm^2^ with a γ-dose of approximately 29 MGy. Temperature and pressure inside the ampoule were monitored using the LIANA diagnostic system. The second 21-day irradiation campaign was performed at the WWR-K reactor [4], at a thermal power of 6 MW, during which the EAD operated at 170–190 °C under cooling with primary reactor water. The sensors accumulated the fast-neutron fluence of 1.8 × 10^20^ n/cm^2^, the total neutron fluence of 4.8 × 10^20^ n/cm^2^, and the γ-dose of 2.3 GGy. Temperature and pressure were recorded using the CIRRA diagnostic system. The irradiation parameters for both campaigns are summarized in Table 1.

## 3. Experimental Samples, Methods, and Procedures

Upon completion of the irradiation campaigns [4,22] and subsequent decay storage, the FBG-based sensors were subjected to a series of post-irradiation examination (PIE) procedures aimed at assessing their mechanical integrity and material condition. The investigated set consisted of 12 sensors, including five sensors irradiated at the IVG.1M reactor, six sensors irradiated at the WWR-K reactor, and one unirradiated reference sensor manufactured using the same fabrication procedure (Table 2).

**Figure 3:**
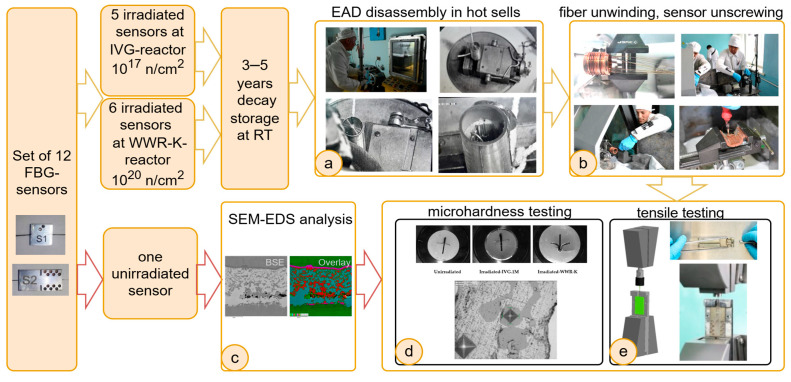
Stages of microstructure investigation and mechanical characterization of FBG-based sensors. (**a**) Disassembly of the experimental ampoule device (EAD) in hot cells after decay storage. (**b**) Fiber unwinding and extraction of the irradiated FBG-based sensors from the irradiation assembly. (**c**) Preparation and examination of the unirradiated reference sensor. (**d**) Vickers microhardness testing of the sensor materials and BFM phases. (**e**) Tensile testing of the capillary-to-plate attachment. The investigated set consisted of five sensors irradiated in the IVG.1M reactor, six sensors irradiated in the WWR-K reactor, and one unirradiated reference sensor.

The irradiated sensors remained inside the experimental ampoule devices (EADs) during a decay-storage period of approximately 3–5 years at room temperature (RT). After this period, the EADs were transferred to hot-cell facilities. The overall sequence of post-irradiation examination and testing procedures is summarized in Figure 3.

### 3.1. Hot-Cell Disassembly and Sensor Extraction

Figure 3a shows photographs of the EAD disassembly process. Before cutting the EADs, the activated region of the EADs corresponding to the segment location was locally shielded with lead blocks. EAD dismantling started with a circumferential cutting of the tube connecting the upper and lower shells. The EAD lower shell containing the segment with the sensors was then cut inside the radiation-shielded cell using a remote copying-type manipulator under visual control through the shielding glass and with video cameras. Before cutting, the EADs were rigidly fixed in a clamping device. Cutting was performed with a 400 mm diameter cutting wheel at a rotational rate below 600 rpm. Upon completion of the cutting procedure, the segment with the sensors and thermocouples was clearly visible inside the EAD.

The segment was then extracted using a specially designed tool equipped with two hooks. After extraction, three thermocouples were cut off and removed, the further operations being focused on the fiber-optic components. At first, the long fiber lengths were unwound from the external surface of the segment (Figure 3b). Fiber unwinding was performed manually behind a 100 mm thick protective screen, the segment being rigidly fixed in a vise. During the unwinding procedure, each fiber was temporarily wound onto a separate reel to preserve the original fiber arrangement.

To extract the FBG-based sensors, the segment was dismantled and divided into two structural parts to provide access to the internal surfaces. Each sensor was unscrewed from the segment internal surface (Figure 3b). All FBG-based sensors were successfully removed without damage and prepared for subsequent post-irradiation examinations.

In disassembling the irradiated IVG.1M EAD, fiber pigtails of sensors S1-117, S2-62, S2-01K, S1-01K, and SP04 (See Table 2 and Figure 5) were unintentionally cut. These sensors were used in the destructive mechanical testing described below. The remaining sensors with intact fiber pigtails were kept for future post-irradiation optical experiments. A similar disassembling procedure was applied to the WWR-K EAD to provide additional sensors for this investigation (Table 2).

### 3.2. Tensile Pull-Off Testing of Capillary

The test object was a capillary attached to one of the steel plates by laser micro-welding. The mechanical strength of the sensor capillary was evaluated using an Instron 5940 series universal testing system (Instron, Norwood, MA, USA) equipped with a 10–50 N high-precision load cell allowing controlled loading of small-scale joints. Each sensor was mounted in custom-designed microgrips (Figure 3e); the metal capillary was fixed in the upper grip, and the outgoing fiber pigtail was secured in a low-damage clamping fixture to avoid premature fiber breakage. Therefore, the mechanical load was applied to the capillary tube, which in turn applied load to the welded and brazed regions of the sensor assembly. The tests were conducted in displacement-controlled mode at 0.1–0.5 mm/min traverse speed, the capillary being loaded with increasing tension until failure. Tensile tests were performed by loading the capillary until failure. The maximum load, load–displacement curve, and failure mode were registered.

It should be noted that because the tensile experiments are destructive, the same sensor samples could not be tested before and after irradiation. Therefore, comparisons were made between irradiated and unirradiated reference samples, both having been fabricated using the same manufacturing procedure.

### 3.3. Metallographic Sample Preparation

**Figure 4:**
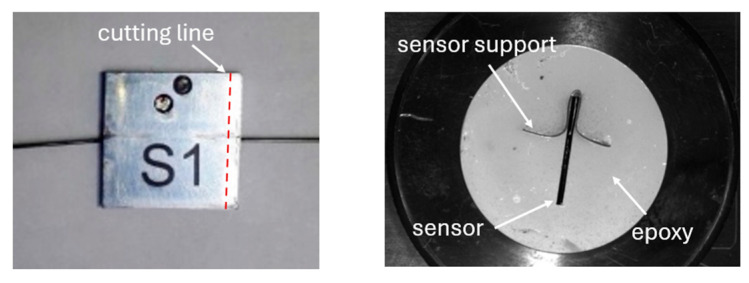
The cutting line on the sensor body photograph and a metallographic sample thus obtained. **Left**: photograph of the unirradiated sensor with the cutting line (dashed). **Right**: the resultant metallographic sample. Thick straight black line is the sensor slice embedded in epoxy resin (white circle).

For the metallographic investigations, the sensor body fragments were cut perpendicular to the fiber using an LCQ-100 and G5000 precision cutting machines (Jinan Hensgrand Instrument Co., Ltd., Jinan, China) equipped with abrasive disks and a water-based coolant. Preparation of the metallographic specimen included embedding the fragments in epoxy resin (Figure 3d), followed by grinding with abrasive papers and final polishing using diamond slurry with particles of decreasing size—3, 1, and 0.25 μm. Figure 4 shows the cutting line on the sensor body photograph and a metallographic sample thus obtained.

To measure radiation-induced changes in the STEMET-1101 BFM, fragments of the bodies of tensile-tested sensors S1-01K and S1-07K were used.

For comparison, the same unirradiated sensor body was also measured.

### 3.4. SEM–EDS Microstructural and Elemental Analysis

Unirradiated sensor S1-11 was used as a reference sample for SEM–EDS analysis. The BFM–steel interfaces and capillary regions were examined using a Tescan Vega3 scanning electron microscope (TESCAN, Brno, Czech Republic) equipped with secondary electron (SE) and backscattered electron (BSE) detectors and coupled with an Oxford Instruments X-Act energy-dispersive X-ray spectroscopy (EDS) system with an SSD detector array (Oxford Instruments NanoAnalysis, High Wycombe, UK) (Figure 3c). SEM imaging was performed in backscattered-electron mode to visualize microstructural changes, porosity formation, and phase segregation induced by neutron–gamma irradiation. The accelerating voltage was 10 kV and the working distance 10–15 mm. The joint morphology, porosity, phase contrast, and possible irradiation-induced microstructural changes were determined with those SEM parameters. Elemental microanalysis, including EDS spectra, maps, and line scans perpendicular to the BFM layer, was carried out at the accelerating voltage of 20 kV and the working distance of 15 mm. These measurements were used to evaluate elemental redistribution, diffusion zones, and possible depletion regions that could influence mechanical integrity of the brazed joint.

### 3.5. Vickers Microhardness Measurements

To evaluate the irradiation-induced changes in the local mechanical properties of the brazed joint, cross-sectional samples were cut from the sensor bodies. Next, the samples were embedded in epoxy, ground, and polished according to standard metallographic procedures. Measurements were conducted using a Qness Q10A+ automatic microhardness tester (ATM Qness GmbH, Golling an der Salzach, Austria) according to the Vickers method with a diamond pyramid indenter at loads of 50 g and 100 g, which was suitable for thin BFM layers and small diffusion zones. Indentations were made in the STEMET-1101 BFM layer, in the heat-affected zone, and in stainless-steel plates of the sensor body to detect possible radiation-induced changes. Microhardness was evaluated for the primary structural phases of the BFM, including the Cu–Sn phase and the Cu–Ni–Fe–P phase, as well as for the sensor plate material. For each analyzed phase and material region, at least 15 individual Vickers indentations were performed, and the microhardness value was calculated as an average.

## 4. Results and Discussion

### 4.1. Tensile Behavior and Failure Modes of Capillary

**Figure 5:**
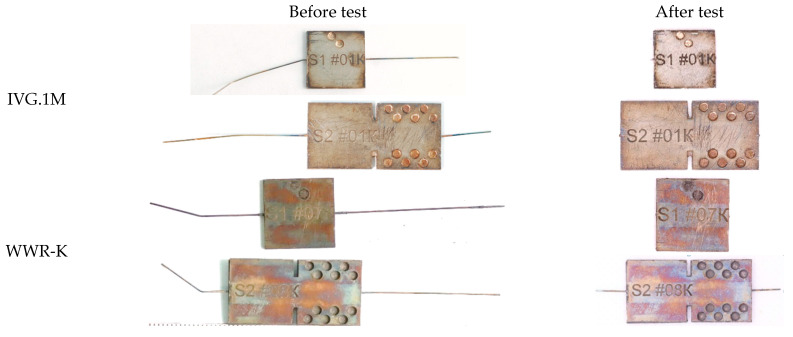
FBG-based sensors irradiated in the IVG.1M and WWR-K reactors before and after the tensile test.

Photographs of the FBG-based sensors irradiated at the IVG.1M and WWR-K reactors before and after the tensile test are shown in Figure 5. The same sensors were then employed for the investigation of the microstructure and microhardness of the sensor metal parts.

**Figure 6:**
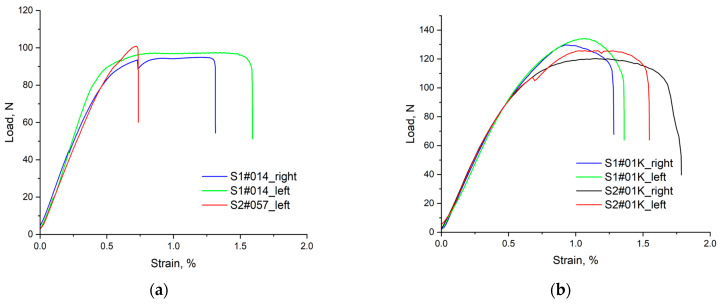

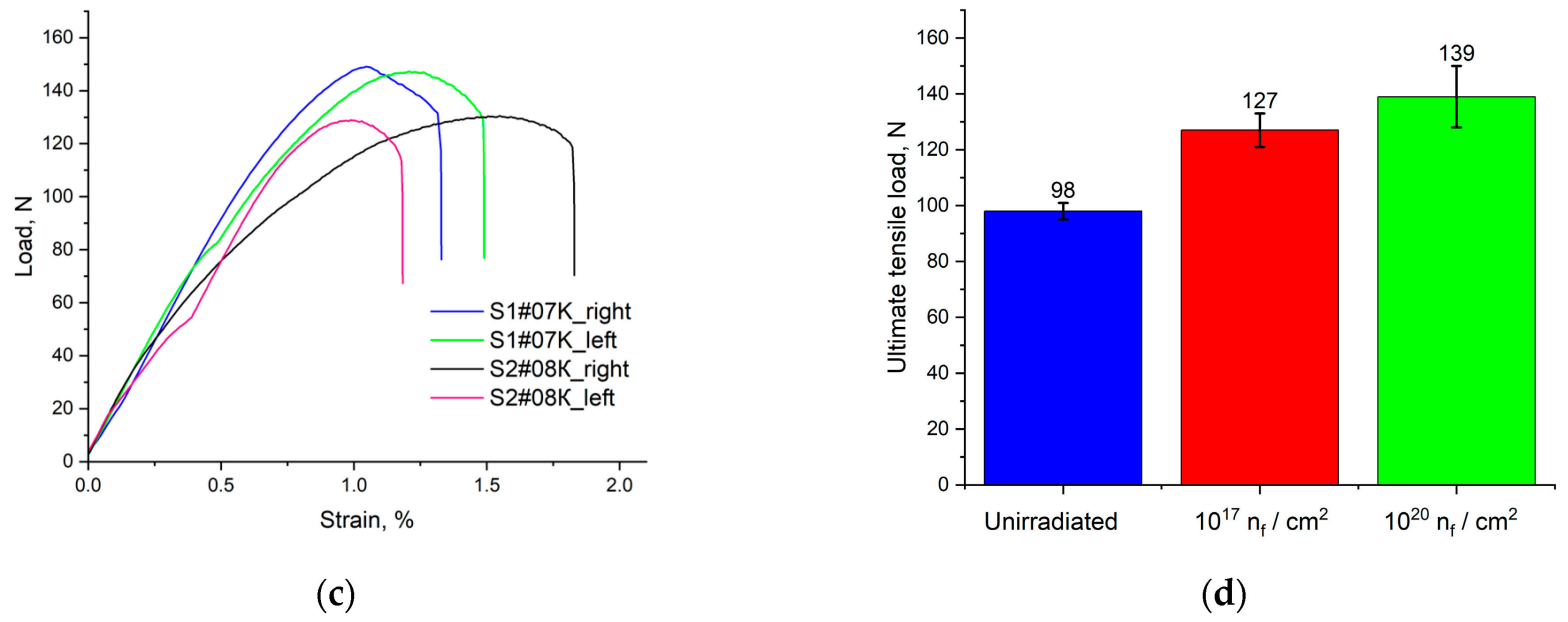
Load–strain curves for the metal-packaged FBG-based sensors: (**a**) unirradiated; (**b**) irradiated at IVG.1M reactor; (**c**) irradiated at WWR-K reactor; (**d**) comparison of the mean values of the ultimate tensile load.

Figure 6 shows the load–strain curves obtained during tests of unirradiated (Figure 6a) and irradiated (Figure 6b,c) sensors. Figure 6d gives a comparison of the mean values of the ultimate tensile load obtained for these sensors.

Note that both the capillary tube and the region where it is attached to the sensor plate were subjected to simultaneous loading. The tensile load was applied directly to the capillary tube, but the load was transferred to the capillary-to-plate attachment region.

In the unirradiated state, the maximum load sustained by the attachment region was virtually comparable to the tensile strength of the capillary tube itself. After irradiation, failure occurred within the capillary tube and at higher loads. Therefore, the observed increase in ultimate load indicates hardening of the capillary tube material, whereas the absence of detachment from the plate indicates that the attachment region conserved sufficient mechanical strength after irradiation.

For interpretation of the tensile-test results, three characteristic failure modes were distinguished. Type 1 corresponds to rupture of the capillary tube away from the laser micro-welded joint; Type 2 corresponds to rupture or fragmentation of the laser micro-welded attachment region; and Type 3 corresponds to partial detachment or damage of the capillary tube near the welded joint due to progressive separation of individual weld spots.

Failure of the unirradiated sensor was mainly due to Type 1 failure, i.e., rupture of the capillary tube away from the welded joint (green curve in Figure 6a), whereas Type 2 failure, i.e., rupture of the laser micro-welded attachment region, was less frequent. In both cases, the ultimate tensile loads proved to be nearly the same (~100 N), indicating comparable load-bearing capacities of the capillary tube and of the welded joint in the unirradiated state. When failure occurred within the capillary tube wall, localized plastic deformation developed, resulting in the formation of a pronounced necking region before rupture. Some load–strain curves exhibited step-like behavior in both elastic and plastic deformation regimes and were classified as Type 3 failure (blue curve in Figure 6a). The steps were associated with the detachment of the most strained laser-welded spots located at the edges of the spot-welded region.

**Figure 9:**
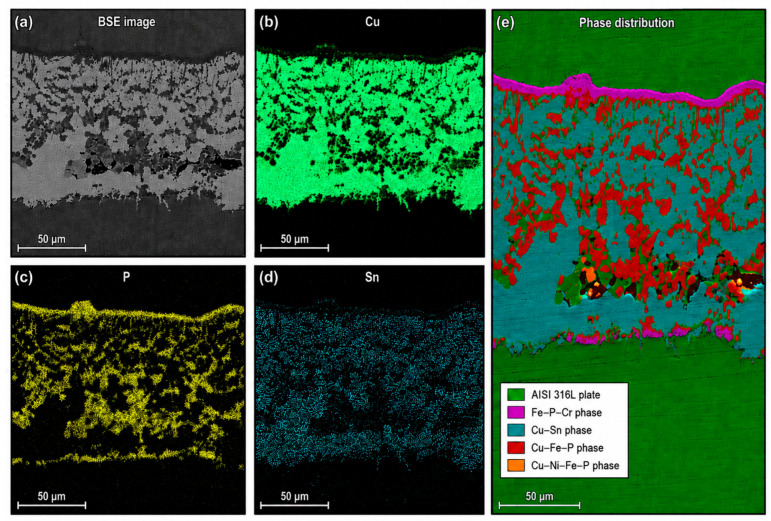
SEM–EDS analysis and phase distribution of the STEMET-1101 brazed region in the FBG sensor: (**a**) BSE image of the brazed joint region; (**b**) Cu elemental map; (**c**) P elemental map; (**d**) Sn elemental map; (**e**) reconstructed distribution of the main phases, including the AISI 316L plate, Fe–P–Cr phase, Cu–Sn phase, Cu–Fe–P phase, and Cu–Ni–Fe–P phase.

In the irradiated sensors, tests revealed a pronounced radiation hardening effect [31]. With increasing radiation dose, the ultimate load increased to reach 127 N for the sensor irradiated at the IVG.1M reactor (Figure 9b) and 139 N for the sensor irradiated at the WWR-K reactor (Figure 6c). In addition, after irradiation, the region of plastic strain became wider along the *Y*-axis.

**Figure 7:**
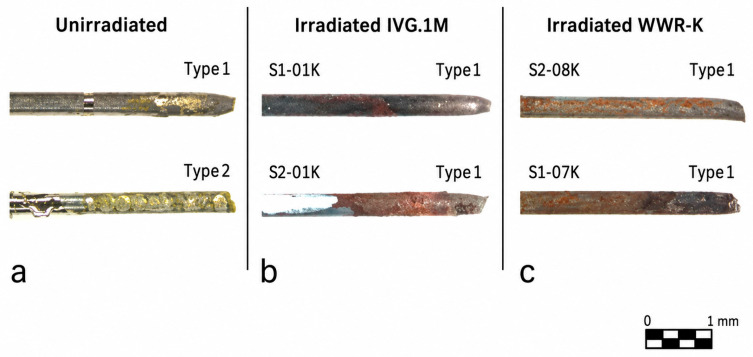
Typical post-test appearance of unirradiated (**a**) and irradiated FBG-based sensors (**b**,**c**) the dominant failure mechanisms observed during tensile testing.

It should be noted that, for all tested irradiated sensors, failure occurred within the capillary tube itself rather than in the capillary-to-plate attachment region (Figure 7). This result primarily indicates irradiation-induced hardening of the capillary tube material. At the same time, the absence of capillary detachment from the stainless-steel plate demonstrates that the attachment region conserved sufficient mechanical strength after irradiation. Thus, the mechanical integrity of the laser-welded capillary–plate joint was not affected by irradiation.

### 4.2. Baseline Microstructure and Phase Constitution of the STEMET-1101 Brazed Joint

The purpose of this section is to describe the initial microstructure and phase composition of the STEMET-1101 brazed joint in the metal-packed FBG-based sensor. Because structural characterization of the irradiated samples was not be performed, the presented microstructural analysis is limited to the unirradiated condition and serves as a reference for interpreting the microhardness and mechanical behavior discussed in the following sections. Moreover, it appears that it is the initial microstructure of the joint that determines mechanical radiation hardness of FBG-based sensors of the design investigated.

**Figure 8:**
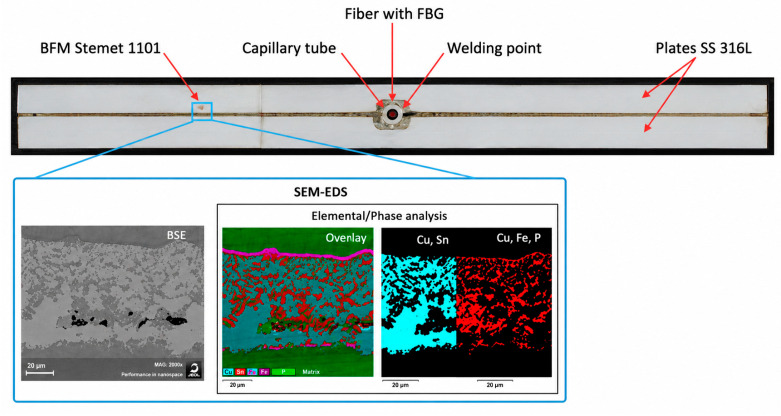
SEM–EDS and microhardness analysis of the sensor body cross-section at the BFM layer. The upper photograph shows a slice of the sensor body made perpendicular to the fiber axis. A blue square indicates the approximate position at which the analysis was performed.

An overview of the sensor assembly and the regions selected for microstructural characterization are shown in Figure 8.

As shown in Figure 9, the STEMET-1101 brazed region exhibits a heterogeneous microstructure formed by mutual diffusion between the filler metal and the 316L stainless-steel plate. No sharp interface is observed; instead, a diffusion zone is formed, indicating good metallurgical bonding and extensive mutual diffusion that occurred during brazing. Based on the selected elemental maps and reconstructed phase distribution, four main phases were identified in the brazed region: a Cu–Sn phase, a Cu–Fe–P phase, a Cu–Ni–Fe–P phase, and an Fe–P–Cr phase (see Table 3). These phases are consistent with previous reports on STEMET-1101 brazing filler metal [30,31]. These studies showed that Cu–Ni–Sn–P-based alloys form multiple distinct phases and diffusion zones, which agrees with our observations.

### 4.3. Phase-Dependent Microhardness Response of Brazed Joint After Irradiation

**Figure 10:**
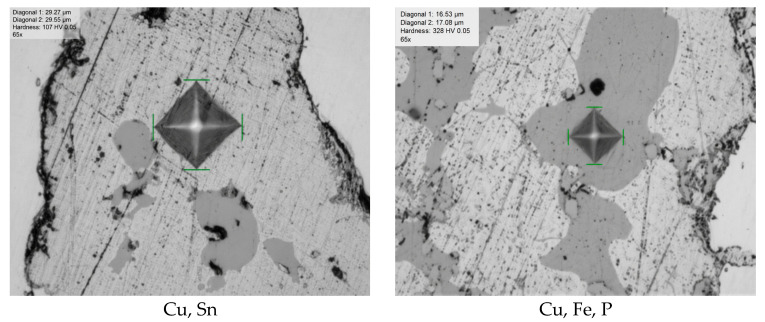
Diamond-pyramid imprints on Cu–Sn and Cu–Fe–P phase particles of STEMET-1101 BFM.

**Figure 11:**
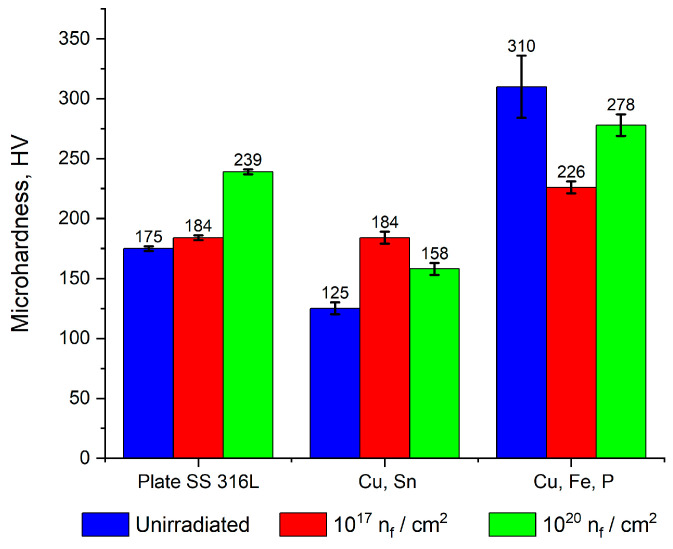
Average microhardness of the STEMET-1101 BFM and of the unirradiated and irradiated sensor plates. Error bars indicate standard deviation. For each phase and material region, n ≥ 15 individual Vickers indentations were used to calculate the average value.

Figure 10 shows diamond-pyramid imprints on individual spots of the Cu–Sn and Cu–Fe–P phases in the STEMET-1101 BFM, whereas Figure 11 shows the average microhardness for the BFM phases and the sensor plates.

For the sensor plates, microhardness lay in the range from 170 to 248 HV, depending on the sample and irradiation history. For the BFM phases, the microhardness values were 125–184 HV for the Cu–Sn phase and 226–310 HV for the Cu–Fe–P phase.

The measurements shown in Figure 11 provide quantitative data on the relative hardness of the different BFM phases and sensor plates before and after irradiation. One can see that the Cu–Fe–P phase was noticeably harder than the Cu–Sn phase in all samples, whereas the sensor plates exhibited intermediate microhardness.

A tendency for radiation-induced hardness variation can be noticed in Figure 11: hardness of the Cu–Sn phase increased after irradiation, whereas hardness of the Cu–Fe–P phase decreased. The sensor plate material (AISI 316L) also exhibited an increased microhardness after irradiation.

The observed changes in microhardness are important because they demonstrate the phase-dependent response of the sensor joint materials to irradiation. The increase in microhardness of the AISI 316L sensor plate is interpreted as evidence of radiation-induced hardening in the austenitic stainless steel. This interpretation is consistent with ref. [32], where it was shown that fast-neutron irradiation of the austenitic stainless steel at about 250 °C leads to hardening associated with the formation and accumulation of irradiation-induced defects that hinder dislocation motion. Such hardening can increase strength but reduces ductility at the same time.

For the STEMET-1101 brazed joint, the response was not uniform because of its multiphase structure. The increase in microhardness of the Cu–Sn phase after irradiation points to radiation-induced aging or defect-assisted hardening. In contrast, the decrease in microhardness of the Cu–Fe–P phase can be associated with radiation-induced destabilization or partial dissolution of initially hardened phases. In this case, redistribution of Fe and P from metastable precipitates back into the copper-based solid solution can reduce the strengthening contribution of this phase.

Therefore, the microhardness measurements were not intended only to compare numerical hardness values, but to identify possible local hardening or softening of individual phases in the brazed joint after irradiation. This data is important for assessing the long-term mechanical reliability of the packaged FBG-based sensor, because local hardening can increase brittleness, whereas local softening can reduce the load-bearing capacity of the individual phases.

The presence of both hardened and softened phases can also increase local mechanical mismatch within the multiphase brazed region. However, the absence of capillary detachment during tensile testing indicates that the sensor package did retain mechanical integrity.

The results obtained should be considered in the context of previous studies on FBG-based sensors and fibers intended for operation in harsh radiation environments. Those studies demonstrated sufficiently high radiation resistance of fibers and FBGs in terms of radiation-induced optical effects (radiation-induced attenuation, spectral changes, silica compaction, radioluminescence, and neutron-induced opalescence [4,5,6,7,8,9,10,11,12,13,22,23,24,25,26,27]). Other studies demonstrated the applicability of FBG-based sensors for temperature and strain measurements in radiation environments, including nuclear-fusion-related systems [1,2,14,15,16,17]. A number of works are devoted to metal cladding, metal packaging, fatigue loading, or embedding of FBG-based sensors, but for applications unrelated to radiation [18,19,20,21]. Those studies did not address any post-irradiation mechanical tests of FBG-based sensor assemblies in metal packaging after high-intensity reactor irradiation. In particular, the mechanical behavior of irradiated capillary fasteners, stainless steel sensor housings, and STEMET-1101 brazed joints remained unstudied. However, this study does provide new experimental data on the mechanical integrity of packaged FBG-based sensors after intense high-neutron-fluence irradiation in a mixed neutron-gamma radiation field.

## 5. Conclusions

Performance of FBG-based temperature and strain sensors before and after high-fluence mixed neutron-gamma irradiation in the IVG.1M (neutron fluence of up to ~10^17^ n/cm^2^) and WWR-K (~10^20^ n/cm^2^) reactors was investigated.

Post-irradiation tensile tests showed that the capillary–plate laser welding joints in the FBG-based sensor body retained their mechanical strength, the ultimate tension loads lying in the range of 120–150 N for most samples. The ultimate load for capillary failure increased with radiation doses to reach 127 and 139 N for samples irradiated at IVG.1M and WWR-K reactors, respectively. Most tensile strength experiments showed that failure of the capillary occurred in the capillary itself, not at the welding joint.

The Cu–Fe–P phase of STEMET-1101 brazing filler metal was found to be noticeably harder than the Cu–Sn phase in all samples, whereas the stainless-steel sensor plates exhibited intermediate hardness. Microhardness measurements of the STEMET-1101 brazing filler metal revealed a radiation-induced hardness increase for the Cu–Sn phase and a decrease for the Cu–Fe–P phase. The sensor plate material (AISI 316L) also exhibited an increased hardness after irradiation. These hardness changes are important because the metallic package transfers strain and temperature to the FBG. Irradiation-induced changes in the mechanical properties of the package may modify the strain-transfer coefficient—a key calibration parameter of the sensor—and consequently affect its long-term measurement accuracy and reliability.

The tested sandwich-like structure of the FBG-based metal-packaged sensor was found to possess sufficient mechanical reliability under irradiation conditions relevant to ITER.

Although no failure of the brazed region was observed during the present experiments, the brazed joint remains the most critical part of the sensor assembly. Direct measurements of irradiated brazed-joint strength and long-term thermo-mechanical stability should therefore be addressed in future studies.

## Figures and Tables

**Table 1:** Irradiation parameters.

Parameter (Units)	IVG.1M [22]	WWR-K [4]
Temperature (°C)	230–330	170–190
Initial pressure (Pa)	~10	0.25
Pressure during irradiation (Pa)	1–100	0.005–0.01
Fast-neutron flux (n/cm^2^·s)	2.4 × 10^13^	1.08 × 10^14^
Fast-neutron fluence (n/cm^2^)	4.5 × 10^17^	1.8 × 10^20^
Total neutron fluence (n/cm^2^)	3.9 × 10^18^	4.8 × 10^20^
γ-dose (Gy)	2.9 × 10^7^	2.3 × 10^9^
Irradiation duration (time)	Hours	21 days
Cooling method	N_2_ flow	Reactor water
Diagnostic system	LIANA	CIRRA

**Table 2:** FBG-based sensors extracted from the EAD (1–11) and a pristine one (12).

No.	Name	Irradiated at Reactor		Notes
		IVG.1M, Φ_E>0.1 MeV_ = 4.46·10^17^ n/cm^2^	WWR-K, Φ_E>0.1 MeV_ = 1.8·10^20^ n/cm^2^	
1	S1-117	•		Sensors had fiber pigtails and were kept for future optical tests
2	S2-62	•		
3	SP04	•		
4	S1-10K		•	
5	S2-01K		•	
6	S2-05K		•	
7	S1-01K	•		Used for tensile tests, micrography, and microhardness tests
8	S2-01K	•		
9	S1-07K		•	
10	S1-18K		•	
11	S2-08K		•	
12	S1-11			Unirradiated sample for SEM-EDS tensile tests, micrography, and microhardness tests

**Table 3:** Chemical composition of the BFM main phases (wt.%).

BFM Phase	Cu	Ni	P	Sn	Fe	Cr
Cu–Sn	89.25	1.34	1.38	3.69	1.56	0.36
Cu–Ni–Fe–P	28.67	27.17	16.51	0.87	19.37	4.32
Cu–Fe–P	66.10	7.54	8.86	1.79	9.03	2.59
Fe–P–Cr	9.98	8.86	18.19	0.70	43.52	15.21

## Data Availability

The original contributions presented in this study are included in the article. Further inquiries can be directed to the corresponding author.

## Abbreviations

The following abbreviations are used in this manuscript:

FBG	Fiber Bragg grating
RIA	Radiation-induced attenuation.
RIL	Radiation-induced luminescence.
RISC	Radiation-induced silica compaction.
NIO	Neutron-induced opalescence.
EAD	Experimental ampoule device.
BFM	Brazing filler metal.
ITER	International Thermonuclear Experimental Reactor.
